# Interpretation bias among breast cancer patients with post-traumatic stress symptoms

**DOI:** 10.3389/fpsyg.2025.1564359

**Published:** 2025-06-18

**Authors:** Miaomiao Wang, Yumin Liu, Yuxi Liu, Bingxue Han, Shuai Teng, Xiaoli Chen

**Affiliations:** ^1^School of Teacher Education, Weifang University, Weifang, China; ^2^Affiliated Hospital of Shandong Second Medical University, Weifang, China; ^3^School of Nursing, Shandong Second Medical University, Weifang, China

**Keywords:** breast cancer, post-traumatic stress symptoms, interpretation bias, cognitive bias, facial expression judgment

## Abstract

**Background:**

This study aimed to investigate interpretation bias in breast cancer patients exhibiting post-traumatic stress symptoms (PTSS), which may affect their cognitive and emotional processing of ambiguous stimuli. Understanding this bias could help inform interventions to address cognitive distortions in this population.

**Methods:**

A total of 234 breast cancer patients were assessed for PTSS using the Impact of Event Scale (IES). Based on their PTSS status, 40 participants were randomly selected from both PTSS-positive and PTSS-negative groups, resulting in 80 participants. All participants completed an ambiguous facial expression judgment task.

**Results:**

The PTSS group showed significantly higher proportions of “sad” judgments and longer reaction times than the non-PTSS group across all levels of facial expression ambiguity, suggesting increased cognitive load when interpreting ambiguous emotional stimuli.

**Conclusion:**

Breast cancer patients with PTSS exhibit a marked negative interpretation bias, which may exacerbate cognitive distortions. These findings highlight the importance of addressing interpretation biases in therapeutic interventions for this population.

## Introduction

Breast cancer is the most prevalent malignancy among women worldwide (Bray et al., 2024), presenting a significant challenge to public health. In China, the incidence rate of breast cancer is increasing at twice the global average, making it the leading cancer among women and a major cause of cancer-related mortality (Qi et al., 2023). Despite substantial improvements in five-year survival rates due to advances in medical treatment, the persistently high incidence and growing number of survivors indicate that breast cancer survivors now constitute a substantial population. Addressing their psychological health and improving their quality of life have thus become important responsibilities for both the healthcare system and society.

A breast cancer diagnosis is a major life event, subjecting patients not only to physical trauma through diagnosis, surgery, chemotherapy, and radiation but also to significant psychological distress. Research has shown that post-traumatic stress disorder (PTSD) is one of the most common psychological issues among breast cancer patients, with prevalence rates ranging from 6.9 to 58% across different studies (Swartzman et al., 2017; Wu et al., 2016). However, more individuals are likely to experience PTSS rather than full PTSD (Li, 2021). PTSS refers to the persistent psychological and physical symptoms experienced by individuals following exposure to significant negative or threatening events. These symptoms may include flashbacks, emotional numbing, trauma-related negative cognitions, and hypervigilance. Studies indicate that as many as 75% of breast cancer patients report subclinical PTSS, even if they do not meet the diagnostic criteria for PTSD (Arnaboldi et al., 2014). Trauma responses adversely affect various aspects of breast cancer patients’ lives, including their quality of life, cognitive function, social interactions, work performance, physical health, and adherence to treatment. Furthermore, these symptoms may exacerbate disease progression, increasing the burden and challenges faced by patients (Mazor et al., 2024; Teng et al., 2022). Given the relatively mild nature of PTSS symptoms, early intervention can effectively mitigate their adverse effects and reduce the likelihood of progression to PTSD.

Ehlers and Clark’s cognitive model of PTSD posits that PTSD develops in individuals who interpret their initial traumatic responses—such as intrusive symptoms and negative emotions—as ongoing threats rather than time-limited experiences (Ehring et al., 2008). For instance, a study of combat veterans found that individuals with PTSD exhibited a higher degree of interpretive bias compared to those without the disorder (Kimble et al., 2002). Similarly, a study of trauma-exposed individuals showed that those diagnosed with PTSD were slower to suppress threat-related interpretations of ambiguous words, consistent with interpretive bias (Amir et al., 2022). Research on victims of interpersonal violence also found that individuals with PTSD symptoms were more likely to interpret ambiguous social situations as threatening compared to a control group (Elwood et al., 2007). While these studies provide strong evidence for a relationship between PTSS and interpretive biases, recent studies have reported no significant correlation between interpretive bias and PTSD symptoms (Deen et al., 2022).

Moreover, the studies mentioned above were not conducted in clinical settings, and their participants differ in significant ways from breast cancer patients. Most breast cancer patients are women, who tend to be emotionally sensitive and reflective. Additionally, the trauma experienced by breast cancer patients is marked by continuity and recurrence. Sources of trauma include the psychological shock of diagnosis, physical and emotional stress during treatment, loss of social roles, and diminished sexual attractiveness due to changes in body image following surgery. Furthermore, the long-term threat of recurrence further exacerbates their psychological distress. Unlike other trauma, the effects of breast cancer-related trauma often persist beyond treatment and may be triggered by specific events such as follow-up exams, symptom recurrence, or cancer progression. Some studies have shown that even factors unrelated to the disease can trigger traumatic responses, negatively impacting patients’ quality of life, social functioning, and intimate relationships (Yahi et al., 2022).

In addition to differences in research participants, the methods used to investigate interpretive biases also require further exploration. Negative interpretive bias refers to the tendency to interpret ambiguous or unclear situations—whether social or non-social—in a negative or threatening manner (Du et al., 2023). Common experimental paradigms for studying interpretive bias include priming tasks, ambiguous situation paradigms, word association tasks, and sentence completion paradigms (Park and Lee, 2023; Schoth and Liossi, 2017). This study intends to use ambiguous emotional facial expressions as the research tool, given the characteristics of the participants. Firstly, text-based paradigms are constrained by factors such as reading ability, imagination, and vision, making them less suitable for individuals with lower educational levels or older age groups. In contrast, facial expression judgment is more universally applicable, operationally feasible, and minimizes confounding factors, thereby reducing experimental error. Additionally, breast cancer survivors often become more attuned to “reading people’s emotions” after their diagnosis, demonstrating heightened sensitivity to others’ facial expressions and evaluations (He et al., 2023; Wang et al., 2024). Research has shown that facial expressions play a crucial role in social interactions, conveying approximately 60% of communicative information, including social evaluative content (Gilboa-Schechtman et al., 2008). Consequently, ambiguous facial expressions are likely to capture the attention of breast cancer patients. Furthermore, emotional expressions in everyday interactions tend to be restrained or subtle, which means that many expressions are unclear or manifest as micro-expressions. These ambiguous expressions often lead to considerable differences in interpretation between individuals. Therefore, interpreting ambiguous expressions is a better measure of individual differences than interpreting clear expressions. Finally, accurately interpreting and responding to others’ facial expressions is essential for social emotional functioning (Bourke et al., 2010; Montagne et al., 2005). Ekman and colleagues have shown that individuals from different cultural backgrounds tend to interpret basic facial expressions similarly (Ekman and Friesen, 1971), suggesting that facial expressions are not significantly influenced by cross-cultural factors, which enhances the generalizability of the findings.

Based on the above analysis, this study hypothesizes that breast cancer patients with post-traumatic stress symptoms (PTSS) exhibit interpretive biases toward ambiguous emotional facial expressions. These biases may play a crucial role in the development of their psychological and behavioral difficulties. Exploring the relationship between PTSS and interpretive bias can provide a theoretical foundation for early intervention, correction of interpretive biases, and mitigation of PTSS symptoms, thereby reducing the risk of PTSD and other mental health problems.

## Methods

### Participants

The sample size was calculated using G*Power 3.1. Based on Cohen (1992) guidelines, a repeated measures analysis of variance (ANOVA) was employed. The parameters were set as follows: between-subjects repeated measures ANOVA; medium effect size (*f* = 0.25); *α* = 0.05; *1-β* = 0.8; number of groups = 2; number of measurements = 9; correlation between repeated measures = 0.5. The calculated total sample size was 72. To account for potential attrition, the final sample size was increased to 80, with 40 participants in each group.

Inclusion criteria:

1. Diagnosis of breast cancer confirmed by biopsy and histopathological examination.
2. Right-handed, with normal or corrected-to-normal vision, and no communication difficulties.
3. Clinically assessed by the attending physician as fit to participate in the study.
4. Aware of their diagnosis and willing to consent to participate.
5. Diagnosis within the past year.

Exclusion criteria:

1. History of other major physical illnesses.
2. History of psychiatric disorders or a family history of psychiatric conditions.
3. History of substance abuse.

A total of 234 participants were recruited. Screening and group allocation were based on the Impact of Event Scale (IES). According to the IES diagnostic criteria, 40 participants (*n_1_*) with a total score greater than 19 were randomly selected for the trauma group, and 40 participants (*n_2_*) with a score below 19 were randomly selected for the non-trauma group.

This study was approved by the Research Ethics Committee of Weifang Medical University (2018YX146). All participants provided written informed consent in compliance with the Declaration of Helsinki. Ethical oversight included continuous monitoring for adherence to protocol throughout the study.

### Measures

#### Post-traumatic stress symptoms

The IES was developed by Horowitz, Wilner, and Alvarez, and later revised by Zhao et al. (2003). The scale consists of two subscales: avoidance symptoms and intrusion symptoms, comprising 15 items, each rated on a 4-point scale. Higher scores indicate more severe post-traumatic stress symptoms. A total score exceeding 19 warrants clinical attention. In this study, the Cronbach’s *α* coefficient for the total scale was 0.81.

#### Emotional stimuli

**Figure 1:**
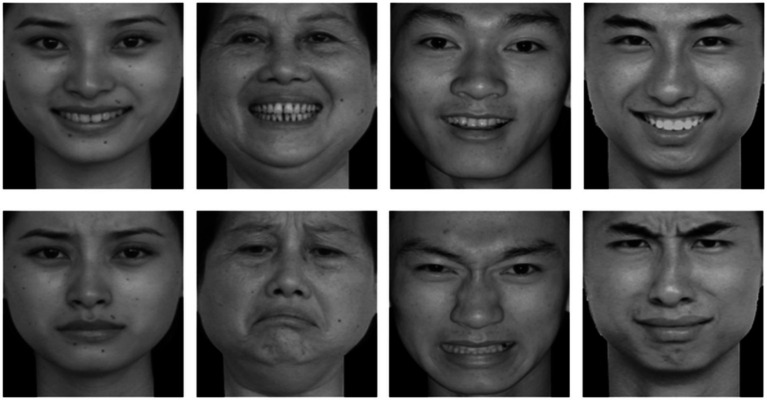
Happy and sad emotional facial expressions of the same participant. Reproduced with permission from Wang and Luo (2005), Chinese Affective Picture System (CAPS).

The emotional facial expressions used in this experiment were selected from the Chinese Affective Picture System (CAPS), developed by Wang and Luo (2005). These images were chosen to exclude irrelevant factors such as hairstyle, facial features, and accessories. To match emotional arousal, one image each of happy and sad expressions was selected from 10 male and 10 female faces, totaling 40 images, as shown in Figure 1.

The happy and sad expressions significantly differed in valence (*t* = 21.30, *p* < 0.001) but not in arousal (*t* = 0.93, *p* = 0.358). No significant differences were observed between male and female images in valence (*t* = −0.66, *p* = 0.517) or arousal (*t* = 1.19, *p* = 0.240) (see Table 1).

**Table 1:** Valence and arousal ratings of different emotional facial expressions.

Image type	Gender	Number (*n*)	Valence	Arousal
Sad	Male	10	2.67 ± 0.28	6.20 ± 1.10
	Female	10	3.23 ± 0.56	5.14 ± 1.52
Happy	Male	10	6.61 ± 0.57	5.97 ± 1.33
	Female	10	6.87 ± 0.63	6.10 ± 0.79

**Figure 2:**
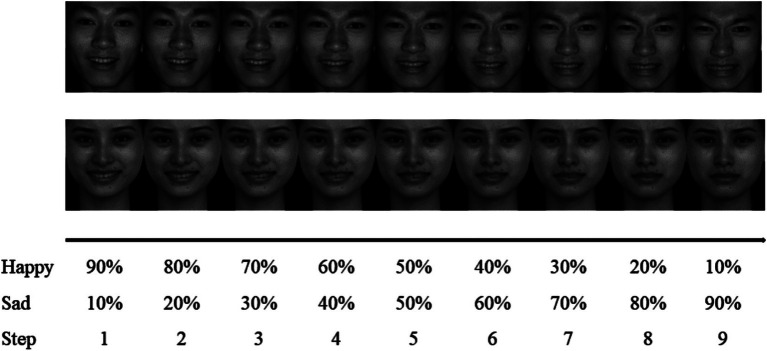
An example of a nine-step morphed sequence. Adapted with permission from Wang and Luo (2005), Chinese Affective Picture System (CAPS).

All images were standardized to a size of 260 × 300 pixels and processed using Photoshop to maintain consistent brightness and contrast. Fanta Morph software was used to generate 11 series by blending the happy and sad expressions of each face, with a 10% variation gradient. The 0 and 100% series, representing clear happy and sad expressions, were excluded. The remaining nine series of blurred emotional facial expressions were used as experimental materials for the blurred facial expression judgment task (see Figure 2).

### Procedure

The experimental environment was kept quiet and free from distractions, with appropriate lighting. The experiment was conducted using a laptop with consistent resolution and brightness, with the screen positioned approximately 70 cm away from the participant. The horizontal visual angle of the emotional facial images was 4.18°, and the vertical visual angle was 4.52°.

The experiment consisted of a practice phase and a formal phase. In the practice phase, each trial began with the presentation of a white “+” symbol at the center of the screen for 500 ms, followed by a blurred emotional facial image for 2,500 ms. Participants were instructed to identify the emotion expressed in the image (using the “J” key for happy and the “F” key for sad). The key assignment was balanced across participants. If no response was made within the allotted time, the image disappeared. After responding, feedback was provided for 1,500 ms (e.g., “You identified the emotion on the presented facial image as sadness”). Participants could proceed to the formal experiment by pressing the “Q” key or return to the practice phase by pressing the “P” key.

**Figure 3:**
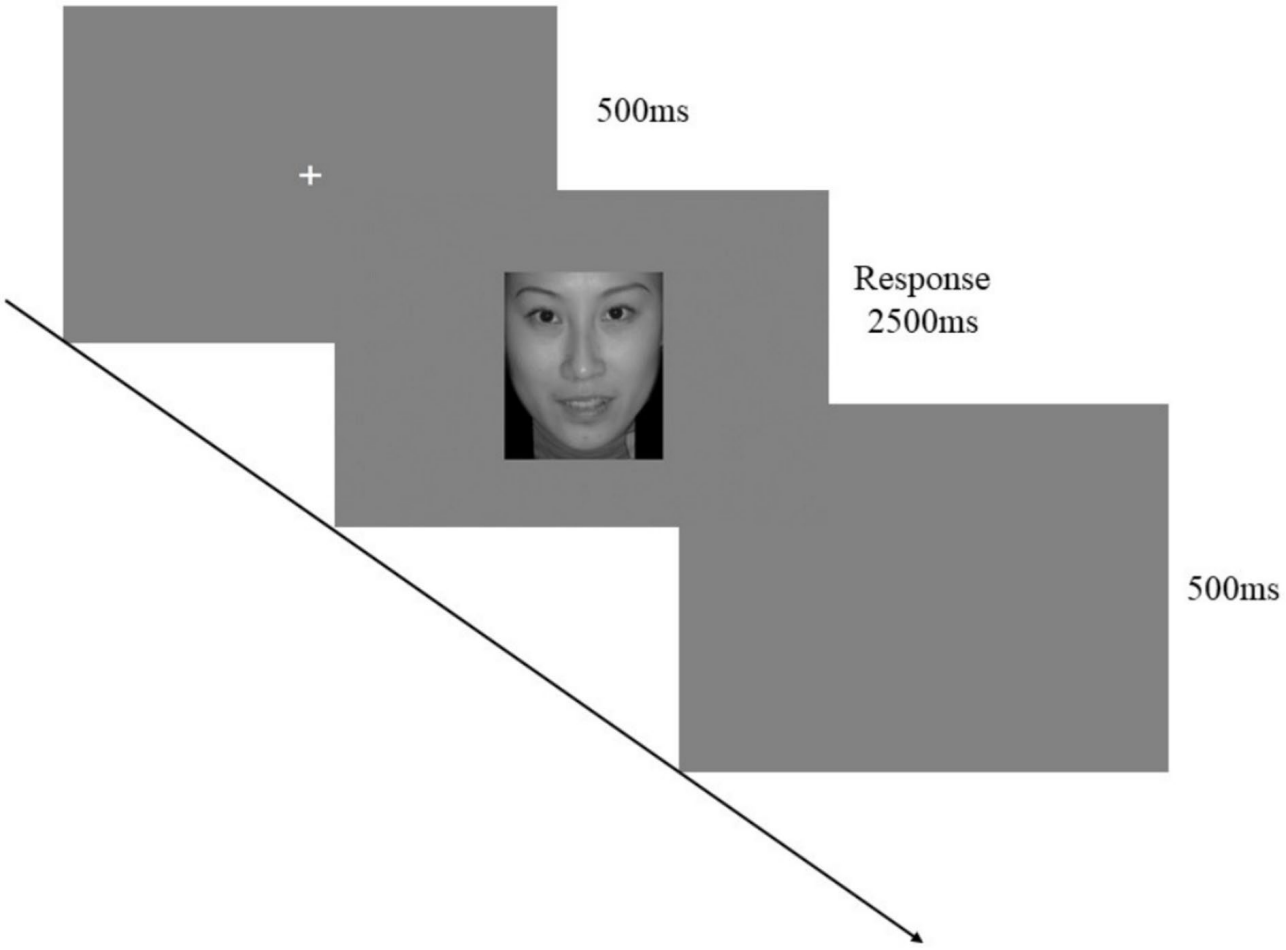
One trial of the ambiguous emotional face recognition task. Adapted with permission from Wang and Luo (2005), Chinese Affective Picture System (CAPS).

The formal phase followed the same procedure as the practice phase, but no feedback was provided. There was a 500 ms blank screen interval between each trial. The experiment included 4 blocks, each consisting of 75 trials, for a total of 300 trials. The proportions of blurred emotional facial images were 40–60%, 50–50%, and 60–40%. The higher blurriness ratios were presented three times per block, while the other ratios were shown once. Breaks were allowed between blocks, and participants could continue by pressing the spacebar (Figure 3).

### Data cleaning and derivation of key variables

Trials without responses were excluded from the dataset. For each participant, the mean and standard deviation of reaction times were calculated, and trials with reaction times exceeding ±3 standard deviations from the mean were removed as outliers. Subsequently, emotional judgments were categorized as either “happy” or “sad.” For each level of emotional ambiguity, the proportion of “sad” responses was computed per participant, serving as an index of negative interpretation bias.

Mean reaction times for each ambiguity level were also calculated for each participant. These two metrics—proportion of “sad” judgments and average reaction times—served as the primary outcome variables in the subsequent statistical analyses.

### Experimental design

A 2 × 9 mixed design was employed, with group (PTSS vs. non-PTSS) as the between-subjects variable and emotional face ambiguity level (nine levels) as the within-subjects variable. The dependent variables included the proportion of “sad” judgments and reaction times at each ambiguity level.

### Statistical analysis

Statistical analyses were performed using SPSS 21.0, including *χ*^2^ tests, independent sample *t*-tests, and repeated measures ANOVA.

## Results

### Sample characteristics

Table 2 presents the demographic and clinical characteristics of participants in the PTSS and non-PTSS groups. The two groups were comparable across all baseline variables, including age, education level, monthly income, marital status, parenthood, time since diagnosis, tumor stage, and type of surgery (all *ps* > 0.05), indicating successful matching.

**Table 2:** Demographic, clinical characteristics and PTSS scores of participants.

Variables		PTSS (*n*_1_ = 40)	Non-PTSS (*n*_2_ = 40)	*χ^2^*/*t*	*p*
Age (years)	30–39	3	4	1.31	1.000
	40–49	10	9		
	50–59	19	19		
	60–69	8	7		
	70–79	0	1		
Education	Primary school	15	10	1.71	0.659
	Middle school	14	18		
	High school	8	8		
	College and higher	3	4		
Occupation status	Employed	7	7	-	-
	Unemployed	28	28		
	Retired	5	5		
Monthly income (CNY)	<2,000	11	3	5.94	0.123
	2,000–3,999	9	10		
	4,000–6,000	11	17		
	>6,000	9	10		
Marital status	Married/cohabiting	39	39	-	-
	Single/divorced /separated/widowed	1	1		
Children	Yes	40	39	-	-
	No	0	1		
Time since diagnosis (months)	<1	1	0	2.26	0.734
	1–3	17	20		
	4–6	11	8		
	7–9	8	7		
	10–12	3	5		
Tumor stage	I	7	7	0.41	0.969
	II	22	24		
	III	5	4		
	IV	6	5		
Types of operations	Lumpectomy	0	5	6.11	0.110
	Mastectomy	8	9		
	Modified radical mastectomy	27	20		
	No surgery	5	6		
IES (*M ± SD*)		31.78 ± 11.4	12.43 ± 4.61	9.95	<0.001

Participants were predominantly aged 50–59 years, accounting for nearly half of each group. Most had completed only primary or middle school and were unemployed or retired. The vast majority were married or cohabiting and had at least one child. Approximately 85% of participants had undergone surgery, with modified radical mastectomy being the most common procedure.

The only significant between-group difference was observed in IES scores. The PTSS group reported significantly higher scores than the non-PTSS group, confirming the validity of the group classification based on PTSS symptom severity.

### Negative interpretation bias

The Mauchly test indicated a violation of the sphericity assumption (*χ*^2^ = 415.94, *p* < 0.001). Greenhouse–Geisser and Huynh-Feldt corrections yielded epsilon (*ε*) values of 0.34 and 0.36, respectively, with smaller ε values indicating a greater degree of violation. Therefore, Pillai’s Trace results from multivariate ANOVA are reported. The results showed a significant main effect of emotional ambiguity level [*F*(8, 71) = 877.22, *p* < 0.001, partial *η^2^* = 0.99], a significant main effect of group [*F*(1, 78) = 80.95, *p* < 0.001, partial *η^2^* = 0.51], and a significant interaction between group and emotional ambiguity level [*F*(8, 71) = 11.89, *p* < 0.001, partial *η^2^* = 0.57]. Simple effect analyses revealed that at each level of emotional ambiguity, the trauma group made a significantly higher proportion of “sad” judgments than the non-trauma group, with the largest differences observed at moderate levels of emotional ambiguity (levels 4, 5, and 6), as shown in Table 3.

**Table 3:** Differences in proportions of “sad” judgments between the trauma and non-trauma groups across emotional ambiguity levels ( $\bar{x}$ *±s*/%).

Level	PTSS (*n*_1_ = 40)	Non-PTSS (*n*_2_ = 40)	Mean difference	*F*	*p*	Partial *η^2^*
1	7.87 ± 7.46	2.00 ± 3.89	0.06	19.46	<0.001	0.20
2	12.08 ± 12.54	3.17 ± 6.71	0.09	15.70	<0.001	0.17
3	18.71 ± 15.20	7.26 ± 8.17	0.12	17.63	<0.001	0.18
4	37.11 ± 15.04	16.03 ± 9.39	0.21	56.52	<0.001	0.42
5	61.60 ± 13.05	34.71 ± 10.16	0.27	105.73	<0.001	0.58
6	79.46 ± 9.67	60.43 ± 13.25	0.19	53.83	<0.001	0.41
7	88.82 ± 10.34	81.56 ± 11.92	0.07	8.45	0.005	0.10
8	94.10 ± 8.73	87.95 ± 12.77	0.06	6.31	0.014	0.07
9	94.74 ± 5.81	90.52 ± 10.56	0.04	4.89	0.030	0.06

### Reaction time differences

The Mauchly test indicated a violation of the sphericity assumption (*χ*^2^ = 232.25, *p* < 0.001). Greenhouse–Geisser and Huynh-Feldt corrections yielded epsilon (*ε*) values of 0.53 and 0.57. Thus, Pillai’s Trace results from multivariate ANOVA are reported. The results indicated a significant main effect of emotional ambiguity level [*F*(8, 71) = 33.88, *p* < 0.001, partial *η*^2^ = 0.79], a significant main effect of group [*F*(1, 78) = 38.71, *p* < 0.001, partial *η*^2^ = 0.33], and a significant interaction between group and emotional ambiguity level [*F*(8, 71) = 3.43, *p* = 0.002, partial *η^2^* = 0.28]. Simple effect analyses showed that at all levels of emotional ambiguity, the trauma group exhibited significantly longer reaction times than the non-trauma group, as summarized in Table 4.

**Table 4:** Reaction time differences between the trauma and non-trauma groups across emotional ambiguity levels ( $\bar{x}$ *±s*/ms).

Level	PTSS (*n*_1_ = 40)	Non-PTSS (*n*_2_ = 40)	Mean difference	*F*	*p*	Partial *η^2^*
1	955 ± 231	667 ± 106	288	51.16	<0.001	0.40
2	970 ± 248	684 ± 110	286	44.46	<0.001	0.36
3	1,043 ± 248	742 ± 120	302	47.94	<0.001	0.38
4	1,091 ± 217	802 ± 140	289	50.16	<0.001	0.39
5	1,109 ± 216	865 ± 150	244	34.43	<0.001	0.31
6	1,066 ± 217	872 ± 154	194	21.14	<0.001	0.21
7	996 ± 209	825 ± 131	171	19.39	<0.001	0.20
8	977 ± 238	794 ± 130	183	18.22	<0.001	0.19
9	946 ± 219	774 ± 114	172	19.40	<0.001	0.20

## Discussion

### Analysis of group differences in proportions of judgments on ambiguous emotional faces

This study revealed significant differences between the two groups in their judgments of ambiguous emotional faces, with the trauma group showing a stronger tendency to classify faces as “sad.” This finding suggests that breast cancer patients with PTSS exhibit a negative interpretive bias toward ambiguous emotional information. Previous studies have demonstrated that individuals with PTSS are often more sensitive to threatening stimuli, interpreting neutral or ambiguous information as threat-related cues (Ehring et al., 2008). For example, breast cancer patients with high levels of fear of recurrence are more likely to interpret ambiguous information as indicative of health threats and report greater pain symptoms. Even after controlling for established predictors such as metacognition and maladaptive thinking, interpretive bias remains a significant predictor of fear of recurrence (Pradhan et al., 2021). Similarly, studies have shown that cancer patients with persistent high-anxiety trajectories exhibit higher levels of interpretive bias (Lam et al., 2018).

In an intervention study, cognitive bias modification training (CBMT) was applied to 120 breast cancer patients to reduce their attention to cancer-related stimuli and to encourage non-threatening interpretations of ambiguous stimuli. The intervention group showed significant reductions in threat-related interpretive bias and cancer-related worry compared to the placebo group (Lichtenthal et al., 2017).

Overall, the findings of this study align with previous research, further supporting the critical role of interpretive bias in the emotional information processing of breast cancer patients with PTSS. These results provide a theoretical foundation for psychological interventions targeting this population, particularly those that aim to reduce negative interpretations through cognitive bias modification. Future research should investigate the neural mechanisms underlying interpretive bias and explore how different types of trauma may differentially influence this bias.

As the two groups were comparable in demographic and clinical characteristics, the differences observed are unlikely to be confounded by background variables, reinforcing the central role of PTSS in shaping cognitive-emotional responses to ambiguous stimuli.

### Analysis of group differences in reaction times

Analysis of reaction times revealed that participants in the trauma group had significantly longer reaction times compared to the non-trauma group across all levels of ambiguity. This finding contrasts with the results of Dai (2011), who investigated negative interpretive bias in elderly individuals with and without depressive symptoms using similar experimental materials and paradigms. Dai found that while greater ambiguity increased response times for both groups, there were no significant differences in reaction times within the same level of ambiguity.

This discrepancy may be attributed to the unique cognitive processing characteristics and emotional regulation challenges of individuals with PTSS. Breast cancer patients with PTSS are more likely to rely on expressive suppression rather than cognitive reappraisal when regulating emotions (Teng et al., 2022). Furthermore, they often exhibit avoidance of negative and positive stimuli, with difficulty disengaging from positive stimuli—factors strongly associated with elevated levels of anxiety, depression, and stress (Han et al., 2024). Research by Pradhan et al. (2021) found that cancer patients, compared to non-cancer controls, show heightened attentional biases toward salient stimuli (e.g., cues of vigilance), particularly among those with higher anxiety levels.

The cancer threat interpretation model posits that attentional bias increases patients’ monitoring of bodily symptoms, while interpretive bias leads them to perceive these symptoms as threatening. These biases reinforce one another, eliciting emotional responses such as anxiety, fear, and worry, which in turn amplify physical symptoms like pain, creating a vicious cycle (Heathcote and Eccleston, 2017). These findings suggest a bidirectional relationship between PTSS and interpretive bias: interpretive bias amplifies individuals’ negative interpretations of trauma, while PTSS symptoms further exacerbate interpretive bias. This interaction may explain the trauma group’s heightened vigilance and threat evaluation tendencies, resulting in longer reaction times.

### Clinical implications

This study offers new insights into the cognitive-affective profiles of breast cancer patients with PTSS, highlighting interpretation bias as a potential target for early psychological intervention. Techniques such as cognitive bias modification training (CBMT), cognitive restructuring, and mindfulness-based therapies may help interrupt the feedback loop between biased interpretations and emotional distress, potentially mitigating the risk of developing chronic post-traumatic symptoms.

The sample in this study primarily consisted of middle-aged women with relatively low educational and income levels, a demographic profile commonly seen in public hospitals and regional cancer centers in China. While this enhances the ecological validity of our findings within similar clinical populations, caution is warranted when generalizing the results to younger, higher-income, or culturally diverse groups. Future studies should aim to replicate these findings in broader, more heterogeneous samples to examine their cross-cultural applicability.

Moreover, the use of culturally adapted ambiguous facial stimuli in this study demonstrated strong ecological relevance, supporting their utility in emotion processing research across clinical populations.

### Limitations of the study

Despite its meaningful findings, this study has several limitations. First, the sample was drawn from a single medical institution, introducing potential selection bias. The generalizability of the findings requires further validation. Future studies should expand the sample to include breast cancer patients from diverse geographic and cultural backgrounds. Second, the cross-sectional design precludes causal inferences about the relationship between interpretive bias and PTSS. Longitudinal or intervention-based studies are needed to explore the dynamic interplay between these factors.

Third, while the experimental paradigm using ambiguous emotional faces effectively controlled for certain confounding variables, its ecological validity is limited. The paradigm may not fully capture patients’ responses in real-world social contexts. Future research could leverage virtual reality technologies to simulate more life-like scenarios, enhancing the external validity of the findings.

## Data Availability

The raw data supporting the conclusions of this article will be made available by the authors without undue reservation.
